# Micro-Pillar Integrated Dissolving Microneedles for Enhanced Transdermal Drug Delivery

**DOI:** 10.3390/pharmaceutics11080402

**Published:** 2019-08-10

**Authors:** Seunghee Lee, Shayan Fakhraei Lahiji, Jeesu Jang, Mingyu Jang, Hyungil Jung

**Affiliations:** 1 Department of Biotechnology, Building 123, Yonsei University, 50 Yonsei-ro, Seodaemun-gu, Seoul 03722, Korea; 2 Juvic Biotech, Inc., No. 208, Digital-ro 272, Guro-gu, Seoul 08389, Korea

**Keywords:** micro-pillar integrated microneedle, microneedle applicator, transdermal drug delivery, dissolving microneedle, transdermal delivery enhancement

## Abstract

The dissolving microneedle (DMN) patch is a transdermal delivery system, containing arrays of micro-sized polymeric needles capable of encapsulating therapeutic drugs within their matrix and releasing them into the skin. However, the elastic properties of the skin prevent DMNs from complete insertion and accurate delivery of encapsulated compounds into the skin. Moreover, the adhesive materials used in patches may cause skin irritation, inflammation, and redness. Therefore, we developed a patchless, micro-pillar integrated DMN (P-DMN) that is simple to fabricate and enhances transdermal drug delivery compared with traditional DMN patches. The micro-pillars were made of polymethyl methacrylate at a height of 300 μm and a base diameter of 500 μm. To fabricate P-DMNs, we employed hyaluronic acid, which is a widely used derma filler and plays a role in tissue re-epithelialization. We demonstrate that utilizing P-DMNs significantly improves the delivery efficiency of an encapsulated drug surrogate (91.83% ± 7.75%) compared with traditional DMNs (64.86% ± 8.17%). Interestingly, P-DMNs remarkably increase the skin penetration accuracy rate of encapsulated drugs, up to 97.78% ± 2.22%, compared with 44.44% ± 7.85% in traditional DMNs. Our findings suggest that P-DMNs could serve as a highly accurate and efficient platform for transdermal delivery of various types of micro- and macro-biomolecules.

## 1. Introduction

Among the available drug delivery routes, medications are generally administered orally or through hypodermic injection [1,2]. While oral administration is a patient-friendly route, the bioavailability of encapsulated pharmaceuticals is affected by the first-pass effect of the body [3,4,5]. Alternately, hypodermic injection is advantageous over oral delivery, as it delivers drugs directly into the bloodstream, enabling a highly accurate delivery [1,6,7]. However, the limitations of hypodermic injection include injection pain, the requirement of expertise to administer an injection, and the potential for infections caused by needle stick waste [8,9]. Transdermal delivery, on the other hand, is regarded as an alternative route for the delivery of compounds with a molecular weight of ≤500 dalton [10]. Although various chemical enhancers and nanosystems, including nanoemulsions and nanoparticles, have been developed to improve the transdermal delivery of drugs, achieving a precise delivery dosage is still a challenge, due to the barrier properties of the skin [11,12,13]. Therefore, dissolving microneedles (DMNs) were developed as a patient-friendly administration system to overcome the above-mentioned obstacles [14,15]. DMNs are polymeric, micro-sized needles capable of encapsulating medicines within their matrix and releasing them upon application for both local and systemic delivery [16,17]. As DMNs are remarkably smaller than traditional hypodermic needles, they are considered minimally invasive and are less likely to cause pain upon application, making them a patient-friendly transdermal drug delivery approach [18,19,20,21]. 

Drug-encapsulated DMNs are generally fabricated in arrays onto sticky patches and applied onto the skin for minutes to hours, depending on the type and viscosity of their backbone matrix materials [22,23,24,25]. Fabricating DMNs over patches, however, was shown to affect delivery accuracy and reduce the efficiency of the encapsulated drugs, due to the incomplete penetration of DMNs into the skin [26,27]. The vast majority of studies showed that because of the physico-chemical properties of the skin, DMNs are not completely inserted into it, resulting in a random and inconsistent release of encapsulated compounds with each application [28,29]. Moreover, the adhesive chemicals used in the patches have been shown to cause skin irritation, redness, and inflammation [30]. 

Over the past decade, numerous studies have focused on improving the delivery efficiency of DMNs [31,32,33]. Two-layered and arrowhead DMNs, comprised of a non-drug-loaded polymer layer at the bottom and a drug-encapsulated tip, have been introduced to improve drug delivery efficiency compared with traditional DMNs. Moreover, a recent study introduced a method to fabricate DMNs over metal pillars to increase delivery the accuracy and efficiency of encapsulated drugs [34]. The pillars function as an applicator, to fully insert the DMNs into the skin by equal distribution of the application force onto each DMN. Alternately, pillar-based DMN systems were developed to improve the delivery efficiency of DMNs by mechanically inserting DMNs into the skin without utilizing patches [35]. As the pillars were fabricated with the same or smaller diameters than the DMNs, there was a high risk of skin damage, irritation, redness, and swelling caused by accidental insertion of the pillars into the skin. In addition, the requirement of a complex applicator system may limit the mass production of such systems. Therefore, there is a critical need to develop an economical DMN system that is both simple to fabricate and that can efficiently deliver the encapsulated drug without damaging the skin.

In this study, to overcome the above-mentioned limitations and improve the delivery efficiency of encapsulated agents, a minimally invasive, micro-pillar integrated DMN (P-DMN) system was developed. Micro-pillars were fabricated with a height of 300 μm and a base diameter of 500 μm, in order to ensure that only DMNs and not the pillars penetrate the skin, resulting in a safe application without damaging the skin. Through a series of in vitro and in vivo experiments, we demonstrated that P-DMNs can deliver encapsulated materials with significantly higher efficiency than traditional DMN patches. Importantly, the simple and economical fabrication process for P-DMNs suggests their potential to be employed as a transdermal drug delivery platform in the future.

## 2. Materials and Methods 

### 2.1. Fabrication of P-DMNs

**Figure 1:**
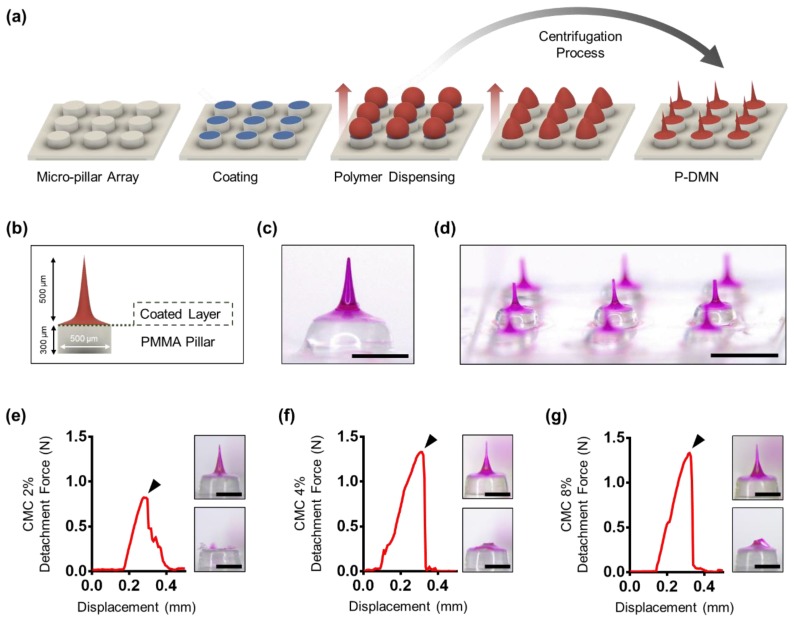
Fabrication of micro-pillar integrated dissolving microneedles (P-DMNs). (**a**) Schematic illustration of the P-DMN fabrication process. First, the micro-pillar array is arranged with a layer of coating. Next, the drug surrogate-encapsulated polymer mixture is dispensed over the micro-pillars, followed by centrifugation to form P-DMNs with a sharp tip. Red arrows indicate the direction of centrifugal force applied to dispensed polymers. (**b**) A P-DMN consists of three layers: a polymethyl methacrylate (PMMA) pillar, a carboxymethylcellulose (CMC) layer, and a dissolving microneedle (DMN). Bright-field microscopy image of (**c**) a single and (**d**) a 3 × 3 array of P-DMNs. (**e**–**g**) Force required to separate a P-DMN fabricated with 2%, 4%, and 8% CMC. Bright-field microscopy images on the right panels show P-DMNs prior to and post-evaluation. Arrows indicate the detachment point from micro-pillars. Scale bars in (**c**,**e**–**g**) are 300 μm, and 1 mm in (**d**).

P-DMNs were fabricated over a 3 × 3 polymethyl methacrylate (PMMA) micro-pillar array (height: 300 μm, diameter: 500 μm), manufactured through the molding technique. First, carboxymethylcellulose (CMC) (2%, 90 kDa, low-viscosity; Sigma, St Louis, MO, USA) was dispensed onto the micro-pillars using an automated X, Y, and Z stage (SHOT mini 100-s, Musashi, Tokyo, Japan), and dried for 5 min producing a thin CMC layer over the array (Figure 1a). To fabricate the DMNs, hyaluronic acid (HA) (32 kDa, Soliance, Pomacle, France), used as the polymeric backbone matrix of the DMNs, was dispensed over the coated pillar array. Rhodamine B (0.3%, 479 Da; Sigma Aldrich) was employed as the drug surrogate. P-DMNs with a height of 500 ± 30 μm were fabricated through the centrifugal lithography, dissolving microneedle fabrication method, by setting the centrifuge force at 400 *g* toward the topmost portion of the dispensed polymers for 1 min (Figure 1b and Figure S1) [36]. During the centrifugal process, the dispensed HA polymer droplets were self-shaped into DMNs with a sharp tip, and solidified for an additional 1 min to achieve a complete evaporation (Figure 1c,d and Figure S2). Scanning electron microscopy (SEM) images were taken using MERLIN SEM equipped with a GEMINI II column (Carl Zeiss, Oberkochen, Germany). The traditional DMNs were fabricated with the same volume of polymer mixture dispensed on the CMC layer and centrifuged at 400 *g* for 1 min. Both P-DMNs and traditional DMNs were fabricated with the same height. 

### 2.2. Separation Force Measurement

The separation force of P-DMN from PMMA was determined using Z0.5 TN (Zwick/Roell, Ulm, Germany) at a speed of 1.0 mm/min. Micro-pillar arrays were coated with a fixed dispensing volume using CMC at 0%, 2%, 4%, and 8% (*w*/*v*). A single P-DMN from each group was positioned horizontally against a sensor probe, and its separation force was measured upon downward movement of probe toward P-DMN.

### 2.3. Skin Surface Damage Evaluation

Skin surface damage effects of micro-pillars fabricated with a height of 300 μm and diameters of 300 and 500 μm were compared. Micro-pillars were pushed toward the skin for 1 min at various forces of 0, 4, 7, 11, and 16 N using the Z0.5 TN machine (Zwick/Roell, Ulm, Germany), at a speed of 1.0 mm/min. The surface of the skin was then imaged using a M165 FC bright-field optical microscope (Leica, Wetzlar, Germany). 

### 2.4. Fracture Force Measurement

The mechanical fracture force was assessed to validate the skin penetration ability of DMNs, using a Z0.5 TN at a speed of 1.0 mm/min. P-DMNs and traditional DMNs were prepared and positioned vertically against a sensor probe. The fracture forces were then measured when the probe was pressed vertically against the DMNs at a force up to 1 N.

### 2.5. Skin Penetration Test

To evaluate the skin penetration ability of the DMNs and P-DMNs, they were applied to pig cadaver skin with a surface area of 2.5 cm^2^ and thickness of 1.0 ± 0.1 mm (Cronex, Seoul, South Korea) for 1 min (*n* = 5/group). DMNs were then removed from the skin, and the surface of the skin was stained with 0.4% (*w*/*v*) trypan blue solution (Sigma Aldrich) for 30 min, and then washed for assessment. Specimens were observed and imaged using an M165 FC bright-field optical microscope. The skin penetration rate was evaluated by counting the number of stained pores on the skin.

### 2.6. Analysis of DMN Residue Post-Application

To measure the volume of DMN residue post-application, P-DMNs and traditional DMNs were applied by Z0.5 TN at 0, 4, 7, 11, and 16 N onto pig cadaver skin with a surface area of 2.5 cm^2^ and thickness of 1.0 ± 0.1 mm (*n* = 5/group). At 10 min post-application, both P-DMNs and traditional DMNs were removed from the skin, and their residues were analyzed using online software (SketchAndClac^TM^). The height and surface area of the DMNs was measured from tip to base.

### 2.7. Evaluation of In Vitro Skin Permeation and Sissolution

The skin permeation was evaluated using a Franz diffusion cell (Hanson, CA, United States), filled with 7 mL of pH 7.4 phosphate buffered saline (PBS) per chamber at 32 ± 2 °C, with a stirrer system set at 250 rpm. Drug surrogate-encapsulated P-DMNs and traditional DMNs were applied for 120 min onto the pig cadaver skin (*n* = 5/group) at 11 N, using a Z0.5 TN. A volume of 1 mL was withdrawn from the receptor compartment, and the intensity of the permeated drug surrogate released from the DMNs was measured using a multimode plate reader (VICTORTM X, PerkinElmer, Waltham, MA, USA) at 5, 10, 30, 60, 90, and 120 min post-application. The data were normalized based on the intensity of DMN arrays that were separately dissolved in a Franz diffusion cell chamber.

The diffusion of the drug surrogate inside the pig cadaver skin treated with P-DMNs and traditional DMNs was evaluated separately at 10, 30, and 60 min using a Franz diffusion cell, as described above. The tissues were then sectioned to assess the permeation pattern and localization of diffusion beneath the skin.

### 2.8. Assessment of In Vivo Skin Permeation

Female ICR mice (seven weeks old) were purchased from Orient Bio (Gyeonggi-do, Korea) and given one week to adapt to the environment. In vivo experiments were approved on 2018.09.10 by the Institutional Animal Care and Use Committee under application number of IACUC-A-201808-768-02. The dorsal skin of the mice was shaved using an electric razor and anesthetized by intraperitoneal injection of Tribromoethanol (Avertin 2.5%). The shaved skin was then gently rinsed and treated with P-DMNs and traditional DMNs for up to 120 min (*n* = 5/group). Next, the DMN-treated regions of the mice skin were cut in 1.5 cm^2^ pieces, embedded into the Franz diffusion cell chamber, and stirred for 120 min. DMN arrays were separately dissolved in the Franz diffusion cell chamber and stirred for 120 min. Samples (1 mL/chamber) were evaluated using the multimode plate reader. The data were normalized based on the intensity of the DMN-dissolved chamber.

### 2.9. Statistical Analysis

Means were compared using Student’s *t*-test or one-way analysis of variance (ANOVA), using GraphPad Prism 6 software. *P*-values of <0.05 were considered significant.

## 3. Results and Discussion

### 3.1. Fabrication of P-DMNs

The fabrication process of P-DMNs consists of two main steps: coating with a thin CMC layer over the micro-pillars, and fabrication of DMNs on the coated array. Rhodamine B, a fluorescence tracer dye widely used to evaluate permeation and diffusion rate, was employed as the drug surrogate [26].

To avoid the tissue damage caused by penetration of micro-pillars into the skin, micro-pillars should be wide enough to only apply the DMNs, but not the micro-pillar itself, inside the skin. Evaluation of tissue damage caused by micro-pillars at different application forces showed that those fabricated with a diameter of 300 μm greatly damaged the skin surface upon applying only 4 N, whereas the 500 μm micro-pillars did not damage the skin when applied with a force of 11 N (Figure S3). Therefore, to minimize the chance of skin damage, P-DMNs were fabricated with a diameter of 500 μm. Since PMMA does not cause irritation upon application onto the skin, it was selected as the material for fabrication of pillars [37]. 

Fabricating DMNs through widely used fabrication techniques, including molding and droplet-borne air blowing, requires a base coating that acts as a supporting layer for the fabrication and application of DMNs. Likewise, as DMNs are fabricated using centrifugal lithography, and the force is applied toward the topmost region of the dispensed mixtures, they should be tightly attached to the pillars throughout the fabrication and application process. Thus, we coated the pillars with a thin layer of CMC, which acts as a connecting layer that strongly holds the dispensed mixtures with the micro-pillars during this fabrication process. To determine the optimal minimum attachment force, we evaluated the fabrication of DMNs over different concentrations of CMC-coated micro-pillars at 0%, 2%, 4%, and 8% (*w*/*v*). As expected, the DMNs without CMC coating (CMC 0%) were not fabricated over pillars. Next, we evaluated the fabrication of DMNs over 2%, 4%, and 8% CMC-coated pillars. The results indicated that DMNs were successfully fabricated in the presence of CMC coating. To evaluate how strongly DMNs were attached to the micro-pillars, we measured the force required to detach a single DMN fabricated over a micro-pillar. We found that the detachment force was 0.80 ± 0.02 N in 2% CMC (Figure 1e), and 1.29 ± 0.10 N in 4% and 8% CMC, with no significant differences (Figure 1f,g). These results indicate that 2% CMC is the optimal concentration of coating required for the fabrication of DMNs.

### 3.2. Measurement of Mechanical Fracture Forces 

**Figure 2:**
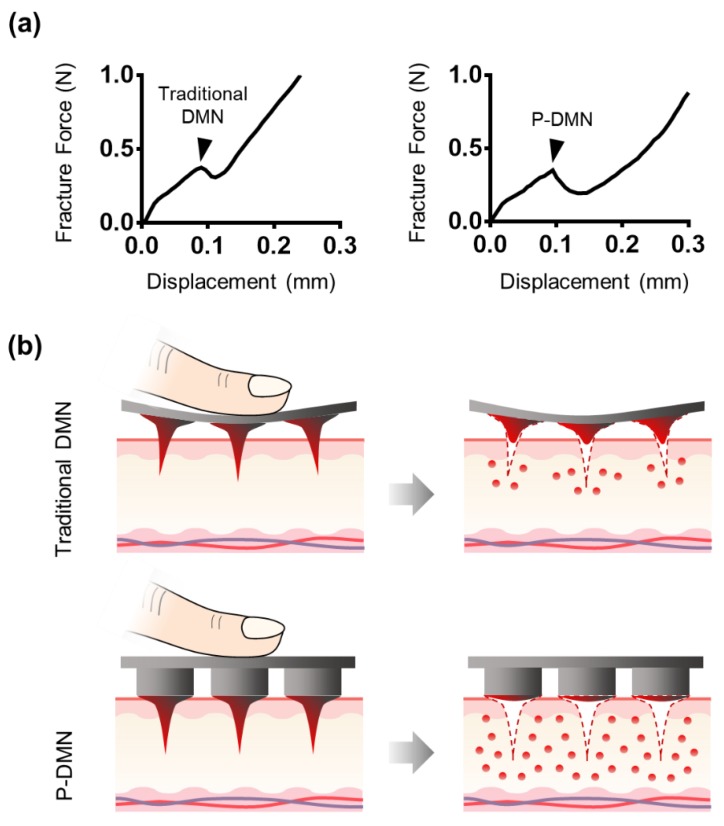

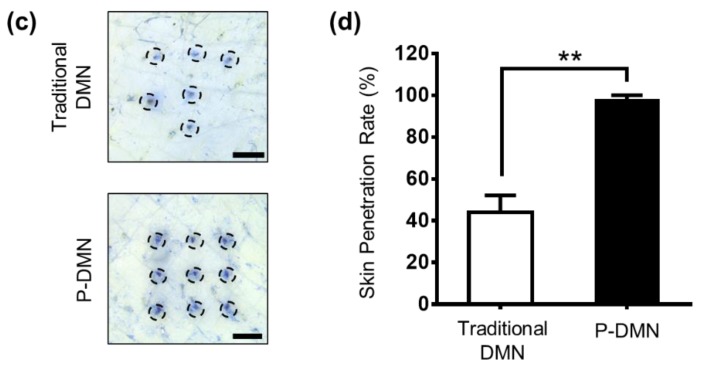
Assessment of mechanical force and penetration of micro-pillar integrated dissolving microneedles (P-DMNs). (**a**) The mechanical fracture force of DMNs confirmed their ability to penetrate the skin without breakage. Arrows indicate the breakage points. (**b**) Illustration of DMN application onto the skin. We hypothesized that unlike in traditional DMNs, the uniform distribution of the application force in P-DMNs would result in an accurate and complete delivery of the encapsulated drugs. (**c**) Skin penetration comparison of DMNs. Traditional DMNs showed an incomplete array of spots, whereas a uniform pattern of 3 × 3 spots was achieved in P-DMN-treated skin. (**d**) Evaluation of skin penetration success rate. Scale bars in (**c**) are 1 mm. ** *p* < 0.01.

As the vast majority of studies have suggested, a minimum force of 0.05 N is required for DMNs to successfully penetrate the skin without breakage. Therefore, we measured the mechanical fracture force of P-DMNs and compared them with traditional DMNs. The fracture force of traditional DMNs and P-DMNs was 0.35 ± 0.02 N and 0.32 ± 0.02 N, respectively (*n* = 5), with no significant differences (Figure 2a). These measurements showed that P-DMNs were completely solidified and capable of skin penetration without breakage, like those in traditional DMN patches. Overall, since both P-DMNs and traditional DMNs were fabricated by using the same polymer mixture with the same geometry, there was no significant difference in their mechanical fracture forces.

### 3.3. Comparison of Skin Penetration Characteristics

Although the mechanical fracture force studies confirmed that DMNs have the required strength to penetrate the skin without breakage, the delivery efficiency of DMNs depends on several factors, including application force and skin elasticity. If the skin is too elastic, DMNs tend to push the skin rather than penetrating and creating pores to deliver the encapsulated drugs. In this study, our hypothesis was that by fabricating DMNs over micro-pillars, it would be possible spread the force onto each DMN, and achieve a uniform and complete insertion of the whole array into the skin (Figure 2b). Thus, we compared the skin penetration characteristics of P-DMNs with traditional DMNs applied onto pig cadaver skin using index finger force (*n* = 5/group). Unlike traditional DMNs, in which DMNs cannot uniformly penetrate the skin, a complete 3 × 3 array of spots was observed on the skin treated with P-DMNs (Figure 2c). Moreover, the penetration success rate of P-DMNs was 97.78% ± 2.22%, which is significantly higher than that of traditional DMNs, at 44.44% ± 7.85% (Figure 2d). These results indicate that P-DMNs could remarkably enhance the penetration accuracy of DMNs compared with traditional DMNs. Moreover, in traditional DMNs, depending on the site of applied force, DMNs were partially inserted into the skin, leading to unequal delivery of the encapsulated drug surrogate within those DMNs. However, micro-pillars distribute the force equally throughout the whole array, resulting in a highly uniform application of every single DMN. It is important to note that in this study, we evaluated the penetration of DMNs fabricated in 3 × 3 arrays, which is a relatively small area, whereas assessing the penetration properties of larger arrays remains to be performed in future studies. Based on the above results, although both DMNs had the same geometry and mechanical strength, we found that utilizing micro-pillars as an integrated applicator could highly improve the skin penetration of DMNs. 

### 3.4. Effect of Application Force on the Dissolution of P-DMNs

**Figure 3:**
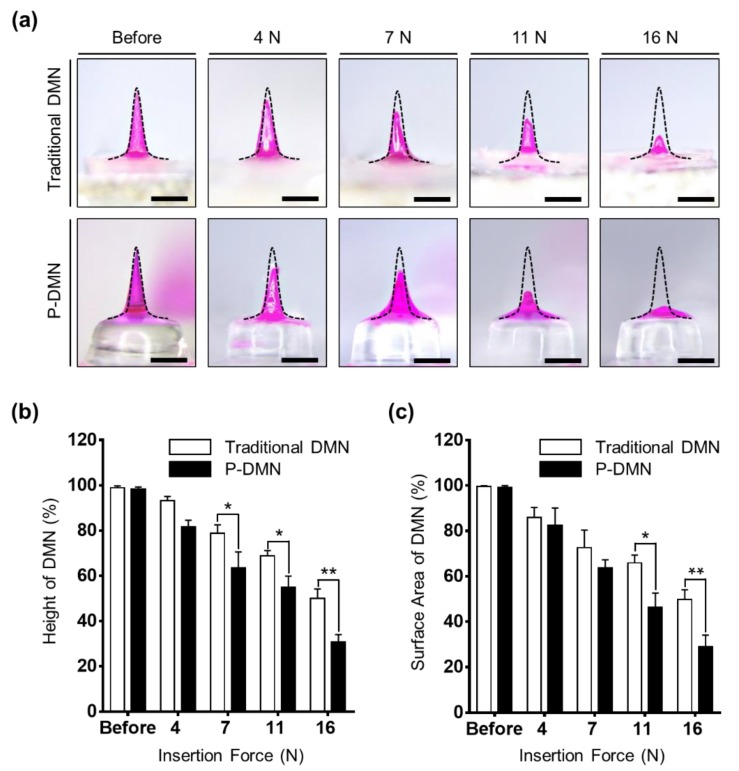
Effect of application force on dissolution of dissolving microneedles (DMNs). (**a**) Comparison of a traditional DMN and a micro-pillar integrated DMN (P-DMN) post-application. Increasing the application force led to increased dissolution of DMNs in both groups. (**b**) Height of DMNs post-application. Increasing the application force from 4 to 16 N reduced the height of the DMNs. (**c**) Surface area changes of the DMNs applied onto the skin at different forces. Similar to the height, the area of DMNs was reduced by increasing the application force. Scale bars in (**a**) are 500 μm. * *p* < 0.05 and ** *p* < 0.01.

The pushing application force for individuals, differing from person to person, plays an important role in penetration accuracy and delivery efficiency of encapsulated drug surrogates within DMNs. Here, we used the index finger to apply DMNs onto the skin. As previously stated, the maximum pushing force of the index finger, affected by gender, age, and application orientation, generally varies between 8 and 14 N [38]. Thus, to find the optimal application force required for P-DMNs for complete skin penetration, we applied them onto pig cadaver skin using an automated machine, with application forces of 4, 7, 11, and 16 N, and evaluated their changes at 10 min post-application (Figure 3a). Both traditional DMNs and P-DMNs were fabricated with the same volume of polymer mixture and the same geometry. Next, the height and surface area changes of the DMNs were measured. The results indicate that at 4 N, there was no significant difference in the height of DMNs in both groups, whereas the height of DMNs was shortened by increasing the application force to 7, 11, and 16 N, suggesting an increased dissolution of the encapsulated drug surrogate. At 11 N, there was 54.89% ± 4.94% of P-DMNs and 68.92% ± 2.24% of traditional DMNs left post-application. Next, we compared the height of DMNs post-application upon applying 16 N and found that 49.94% ± 4.25% of traditional DMNs and 30.83% ± 3.22% of P-DMNs residue was left (Figure 3b). Although an application force of 16 N suggested improved delivery efficiency, as demonstrated earlier, it damages the surface of the skin. Therefore, based on the above findings, we suggested that increasing the application force from 4 to 11 N would significantly improve the delivery efficiency of P-DMNs compared with traditional DMNs. Moreover, at the same application force, P-DMNs were capable of dissolving with a higher efficiency.

Because of the physical properties of the skin, DMNs may bend or break prior to insertion into the skin, leading to inefficient delivery of the encapsulated drugs. Therefore, in addition to the height measurements, the surface area of the DMNs was evaluated, to ensure that the changes in height were solely due to dissolution of DMNs. There were no significant differences in the dissolution of either DMN at application forces of 4 and 7 N. Similar to the data obtained in the height residue experiment, the area of P-DMNs was reduced post-application upon applying 11 and 16 N. At 11 N, while there was 46.36% ± 6.27% of P-DMNs left post-application, a significantly higher area of 65.90% ± 3.48% was measured of traditional DMNs (Figure 3c). As DMNs remained inside the skin only for 10 min, a complete dissolution was not achieved, whereas increasing the application time would result in a remarkably higher release of encapsulated drug surrogates. These analyses suggested that even at the same application force, P-DMNs dissolved a larger volume of the encapsulated drug surrogate compared with traditional DMNs. Moreover, these findings highlight the importance of the applied pushing force on the penetration and delivery of DMN-encapsulated drugs. Altogether, we conclude that an application force 11 N, achieved by the index finger of an adult person, would be optimal to apply P-DMNs and achieve the highest delivery efficiency without damaging the skin. 

### 3.5. In Vitro Skin Permeation and Dissolution Analysis

To evaluate the skin permeation kinetics of drug surrogate-encapsulated DMNs, they were inserted into pig cadaver skin fixed over a Franz diffusion cell for up to 120 min. The permeation and diffusion rate of drugs depend on the chemical properties of the polymeric backbone matrix and its viscosity. Moreover, by modifying the viscosity of the backbone matrix in DMNs, it is possible to achieve a sustained or rapid release of encapsulated drugs inside the skin.

**Figure 4:**
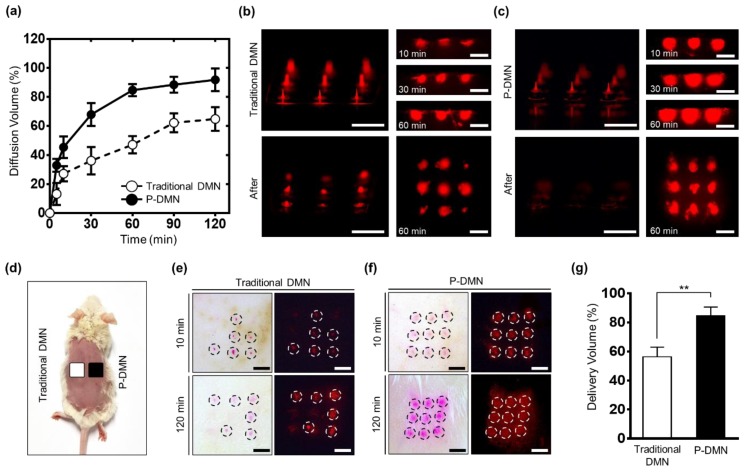
Skin dissolution and permeation evaluation of dissolving microneedles (DMNs). (**a**) Comparison of DMN diffusion pattern in vitro: the micro-pillar integrated DMN (P-DMN) delivered a larger volume compared with traditional DMNs at 120 min. (**b**) At 60 min post-application, a remarkable amount of drug surrogate was left over the patches (left lower panel). (**c**) There was no residue over the micro-pillars at 60 min post-application (left lower panel). The right top panels in (**b**,**c**) show the permeation patterns inside the skin up to 60 min. The right lower panels show the diffusion patterns over the skin surface at 60 min. (**d**) DMNs were applied onto the dorsal skin of mice for up to 120 min. (**e**) The dissolution of the drug surrogate was unequal in the mice treated with traditional DMNs (top panels). At 120 min post-application, the intensity at application spots was further expanded (lower panels). (**f**) The P-DMNs showed a uniform array of spots, indicating an even application (top panels). The intensity of the P-DMNs was remarkably higher than that of the traditional DMNs (lower panels). In (**e**,**f**), the left panels are optical microscopy, and the right panels are fluorescence microscopy. (**g**) In vivo transdermal delivery volume. Scale bars in (**b**,**c**,**e**,**f**), are 3 mm. ** *p* < 0.01.

The results indicate a significantly increased delivery volume in P-DMNs compared with traditional DMNs beginning at only 10 min post-application. The P-DMNs delivered a total volume of 91.83% ± 7.75% at 120 min, while traditional DMNs delivered a remarkably lower amount of 64.86% ± 8.17% (Figure 4a). These findings suggest that P-DMNs could deliver the encapsulated drugs with greater efficiency compared to traditional DMNs. 

The pig cadaver skin was sectioned at 10, 30, and 60 min post-application to visualize the permeation pattern of drug surrogate-encapsulated DMNs. At each point, the extra drug surrogate was removed from the skin surface to detect the volume that had permeated into the skin. At 10 min post-application, the detected intensity of the P-DMN was remarkably higher than that of the traditional DMN. Moreover, although the whole array of P-DMNs could successfully pierce the skin, traditional DMNs failed to uniformly penetrate the skin. At 30 min, while the intensity signal of P-DMN had further increased at each spot, only a slight increase in the intensity of traditional DMNs was detected (Figure 4b,c). While both DMNs were applied with the same force, we observed a significantly higher diffusion in P-DMNs, suggesting a highly accurate insertion of DMNs into the skin. After 60 min of application, unlike in P-DMNs, there was a remarkably high volume of the drug surrogate remaining over the patches in traditional DMNs. Overall, these results confirmed that the P-DMN is capable of delivering the encapsulated drug surrogate with a higher accuracy compared to traditional DMNs. 

### 3.6. Evaluation of In Vivo Skin Permeation 

To examine the penetration and permeation of the drug surrogate in vivo, we applied them onto the shaved dorsal skin of mice for 120 min (Figure 4d). At 10 min post-application, while all P-DMNs successfully pierced the skin and showed a complete array of 3 × 3 spots, the traditional DMNs failed to uniformly penetrate the skin (Figure 4e,f). These results further confirmed our in vitro findings, shown in Figure 2c,d and Figure 4b,c, related to the improved skin penetration success rate of P-DMNs compared with traditional DMNs. Furthermore, at 120 min, a significantly higher intensity was observed for P-DMNs compared with traditional DMNs. Similar to the results of the in vitro evaluations, P-DMNs could deliver the encapsulated drug surrogate with a remarkably higher efficiency compared with traditional DMNs. Importantly, no skin damage or irritation was observed in the mice during the application process up to 120 min.

We next measured the volume of the delivered drug surrogate on each application site. The volume of the drug surrogate delivered by P-DMNs was 84.66% ± 5.96%, which was remarkably higher than that of the traditional DMNs at 56.29% ± 6.71% (Figure 4g). Since the pig cadaver and mice skin possess different physico-chemical properties, such as thickness, lipid content, enzyme activity, and hair follicle density, the delivery volume of the drug surrogate was different in the in vitro evaluations compared with those performed in vivo [39]. Altogether, these findings confirm that utilizing micro-pillars could significantly improve the penetration rate and delivery efficiency of the P-DMN-encapsulated drug surrogate. 

## 4. Conclusions

In this study, the P-DMN was introduced as a novel system to improve the delivery efficiency and accuracy of DMNs compared with traditional DMNs. In brief, the P-DMN is a patchless, micro-pillar integrated DMN applicator that improves the delivery efficiency of drugs encapsulated within DMNs. Our findings confirm that compared with traditional DMNs, P-DMNs significantly improved the delivery efficiency of drugs into the skin by 26.97% ± 7.96% in vitro and 28.37% ± 5.37% in vivo, up to 120 min post-application. Moreover, the application accuracy of DMNs was highly increased in P-DMNs up to 91.83% ± 7.75%, compared with traditional DMNs at 64.86% ± 8.17%. Overall, we assume that the newly developed, applicator-integrated P-DMN could become a promising economical platform for the accurate transdermal delivery of drugs in the future.

## Supplementary Materials

The following are available online at https://www.mdpi.com/1999-4923/11/8/402/s1. Figure S1: Bright-field microscopy images of micro-pillar integrated dissolving microneedle (P-DMN) fabrication, Figure S2: Scanning electron microscopy (SEM) images of micro-pillar integrated dissolving microneedles (P-DMNs), Figure S3: Evaluation of skin surface damage caused by micro-pillars.
