# Supplementary material for: Micro-Pillar Integrated Dissolving Microneedles for Enhanced Transdermal Drug Delivery
Source: Pharmaceutics. 2019 Aug 10;11(8):402. doi: 10.3390/pharmaceutics11080402 (PMC6724014; doi:10.3390/pharmaceutics11080402)
Supplement: Supplementary file 1 [file pharmaceutics-11-00402-s001.pdf]

Supplementary Materials: Micro-pillar Integrated Dissolving Microneedles for Enhanced Transdermal Drug Delivery

Seunghee Lee, Shayan Fakhraei Lahiji, Jeesu Jang, Mingyu Jang and Hyungil Jung

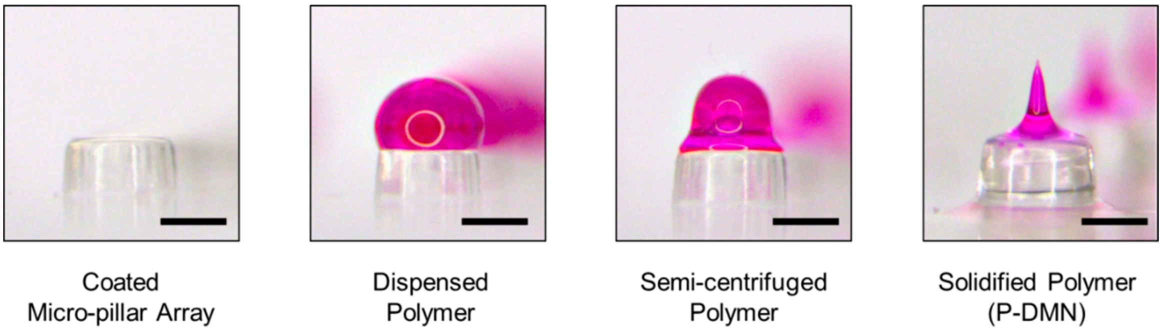

Figure S1. Bright-field microscopy images of micro-pillar integrated dissolving microneedle (P-DMN) fabrication. The micro-pillar array is first coated with a layer of carboxymethylcellulose (CMC). The drug surrogate-encapsulated polymer is then dispensed over the pillar and centrifuged by applying 400 *g* for 1 min. Upon solidification of the polymer, a complete array of P-DMNs was fabricated. Scale bars are 300 µm.

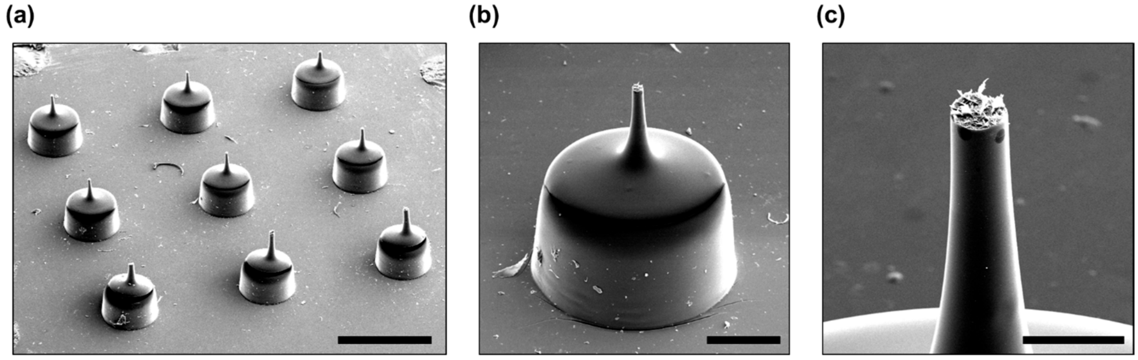

Figure S2. Scanning Electron Microscopy (SEM) images of micro-pillar integrated dissolving microneedles (P-DMNs). (**a**) SEM image of P-DMNs confirmed uniformity of the fabrication throughout the array. (**b**) A single P-DMN. (**c**) Tip portion of P-DMN. Scale bars are 1 mm, 200 µm, and 50 µm, in (a), (b), and (c), respectively.

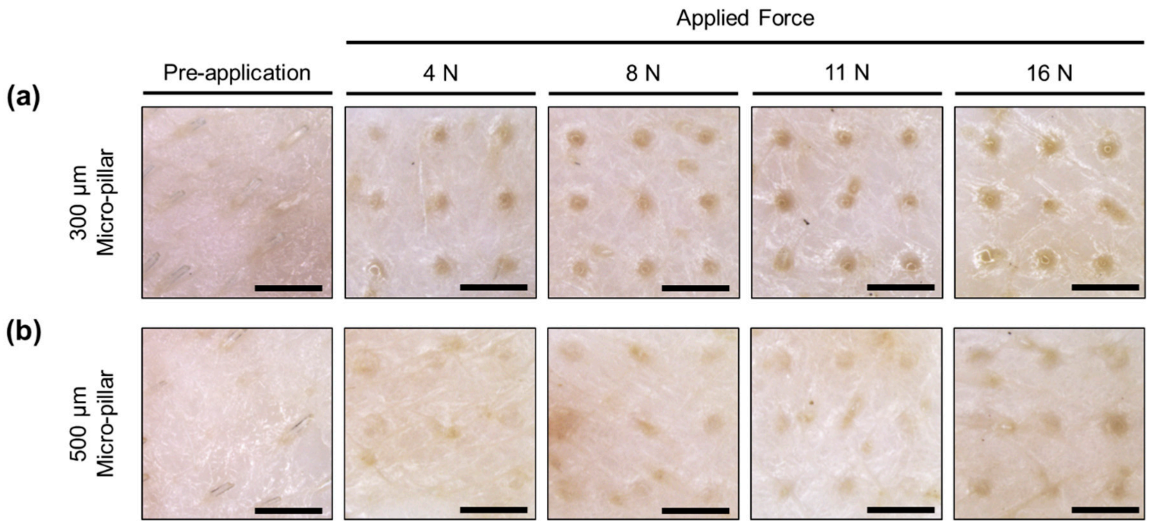

Figure S3. Evaluation of skin surface damage caused by micro-pillars. (**a**) Comparison of skin damage caused by an array of 300 μm micro-pillars showed that upon applying only 4 N, the skin surface was damaged. Increasing the application force up to 16 N further increased the impact of skin damage. (**b**) Because of the wider diameter of the 500 μm micro-pillars, the skin surface was not damaged up to 11 N. Scale bars in (a) and (c) are 1.5 mm.
